# Understanding primary care perspectives on supporting women’s health needs: a qualitative study

**DOI:** 10.3399/BJGP.2023.0141

**Published:** 2023-09-19

**Authors:** Francine Toye, Jennifer MacLellan, Sharon Dixon, Abigail McNiven

**Affiliations:** Physiotherapy Research Unit, Nuffield Orthopaedic Centre, Oxford University Hospitals NHS Foundation Trust, Oxford.; Medical Sociology and Health Experiences Research Group, Nuffield Department of Primary Care Health Sciences, University of Oxford, Oxford, UK.; Medical Sociology and Health Experiences Research Group, Nuffield Department of Primary Care Health Sciences, University of Oxford, Oxford, UK.; Medical Sociology and Health Experiences Research Group, Nuffield Department of Primary Care Health Sciences, University of Oxford, Oxford, UK.

**Keywords:** primary health care, qualitative research, women’s health, general practice

## Abstract

**Background:**

A consultation for the Women’s Health Strategy for England in 2022 highlighted a need to understand and develop how general practice can support women’s health needs.

**Aim:**

To understand the perspectives and experiences of primary care practitioners (PCPs) about supporting women’s healthcare needs.

**Design and setting:**

Interpretive qualitative research set in general practice in England.

**Method:**

PCPs working in general practice settings were recruited through research and professional networks. Semi-structured interviews were conducted via telephone or Microsoft Teams, audiorecorded, transcribed verbatim, and analysed through reflexive thematic analysis.

**Results:**

In total, 46 PCPs were interviewed. Participants had a range of roles and worked in a variety of primary care settings. Results are presented within six themes: 1) being alongside a person from cradle to grave; 2) maintaining the balance between general and specialist skills; 3) generalists and specialists combined make more than the sum of their parts; 4) striving for equity in a collapsing system; 5) firefighting with limited resources; and 6) the GP is being cast as the villain.

**Conclusion:**

The findings show that relationships and advocacy are valued as fundamental for women’s health in general practice, and highlight the adverse impact of threats to these on staff and services. Developing specialist roles and bespoke services can foster staff wellbeing and could support retention. However, care is needed to ensure that service configuration changes do not result in clinician deskilling or rendering services inaccessible. Care is needed when services evolve to ensure that core aspects of general practice are not diminished or devalued. GP teams are well placed to advocate for their patients, including commitment to seeking equitable care, and these skills and specialist knowledge should be actively recognised, valued, and nurtured.

## INTRODUCTION

In England, where general practice is the first port of call, primary care practitioners (PCPs) are essential to women’s health care.^1^^,^^2^ Even when patients are treated in secondary care, they continue their journey in general practice. However, research tends to draw on samples from secondary care.^3^ Women’s health encompasses all health care, although it is often reported in the context of the female reproductive tract. This is emblematised in general practice, which cares for women at all life-course stages. There are gender inequalities, both in women’s health care and the research that underpins it.^4^^,^^5^ Women experience differential health outcomes, for example, in diagnostic time for cancer and in cardiovascular disease.^6^^–^8 The UK’s Women’s Health Strategy for England draws attention to these holistic needs.^9^

From March to June 2021, a consultation for the Women’s Health Strategy for England highlighted a need to understand and develop how general practice can support women’s health needs. Women’s health hubs (‘one stop shops’) were supported by the Women’s Health Strategy,^9^ and although there is an interim evaluation,^10^ no detailed evaluation, to the authors’ knowledge, is available. The consultation highlighted the need to hear women’s voices. The voices of PCPs with experience of delivering services would also help with understanding what works well and the barriers to effective care. The aim of this study was, therefore, to understand the perspectives and experiences of PCPs relating to supporting women’s healthcare needs to inform improved care.

## METHOD

Between March and September 2022, primary care practices across England were sampled through the Clinical Research Network (CRN). Practitioners were self-selecting but population characteristics were targeted to include areas of high health need, diverse ethnicity, and high deprivation. The authors also purposively sampled PCPs with a special interest in women’s health, male GPs, and nurse practitioners. The CRNs contacted practices with an information sheet and potential participants were invited to contact the research team.

**Table:** How this fits in

The Women’s Health Strategy for England highlighted a need to understand and develop how general practice can support women’s health needs. This study’s aim was to hear the voices of primary care practitioners with experience of delivering services, and to further understand what works well to provide quality care. Relationships and advocacy are at the core of general practice and women’s health, and this study highlights threats to these core values and skills. Care is needed when evolving services to ensure that relationship-based longitudinal knowledge of individuals, families, and communities is not devalued, as this is integral to high- quality health and social care.

The topic guide was developed by three authors in response to a perceived gap in the Women’s Health Strategy evidence base, to allow flexibility for the participant to develop and expand on topics of particular importance or relevance to their context (see Supplementary Information S1). The guide was piloted with two practitioners.

Two authors reviewed the topic guide after five interviews. Interviews were audiorecorded and transcribed verbatim, and transcripts checked for accuracy, de-identified, and uploaded to NVivo (version 12) software. Working in collaboration, the research team used the six stages of reflexive thematic analysis to develop themes. This approach is in line with the teams interpretive, non-positivism epistemology, and supported the authors’ aim to explore ‘latent’ (below-the-surface) meaning.

All researchers read the interviews and discussed their interpretations throughout data collection (stage one). One author coded transcripts and suggested provisional themes, supported by narrative, for the research team to discuss (stages two and three). Next, all authors met via Microsoft Teams to develop, review, and refine themes (stages four to six). Analytic collaboration aims to enhance interpretations (rather than reach consensus), and to encourage the researchers to ‘interrogate’ their subjectivity.^11^

The team included experienced qualitative researchers from a range of disciplines: an anthropologist and physiotherapist (the first author), a nurse/midwife (the second author), a GP (the third author), and a social scientist (the senior author). All researchers felt free to discuss, suggest, and challenge each other. When all authors agreed that the themes *‘convey*[ed] *something important’*,^11^ they developed a visual storyline (after several iterations) to convey the ‘story’. Braun and Clark advocate that creativity is central to reflexive thematic analysis, and diagrammatic ‘storytelling’ sits comfortably with this approach.^11^

## RESULTS

In total, 46 (*n* = 41 females) PCPs with 1–30 years primary care experience were interviewed (Table 1). Most were GPs (*n* = 31), 10 with a special interest in women’s health. Nine nurses and six other staff (Table 1) were also interviewed. Interviews lasted from 19–60 min (average 32 min). Thirty PCPs were located within Yorkshire, Humber, and Greater Manchester areas, and 16 within Thames Valley, South Midlands, and London areas.

**Table 1.** Participant characteristics (*N* = 46)

**Role**	**Sex, *n***		**Experience in primary care, years, range (mean)**	**Special interest in women’s health, *n***
	**Male**	**Female**		
**GP**	5	26	1–30 (11)	10
**Nurse**	0	9	3–20 (14)	0
**Other: cardiology technician, clinical pharmacist, healthcare assistant, and nursing associate**	0	6	9–30 (13)	0

The Index of Multiple Deprivation (IMD) indicated that 28 PCPs were located within the 50% most deprived areas in England (with *n* = 11/28 in the 10%–20% most deprived areas), and 15 PCPs within the 10%–30% least deprived areas. IMD data were not included for three PCPs.

**Figure 1.**
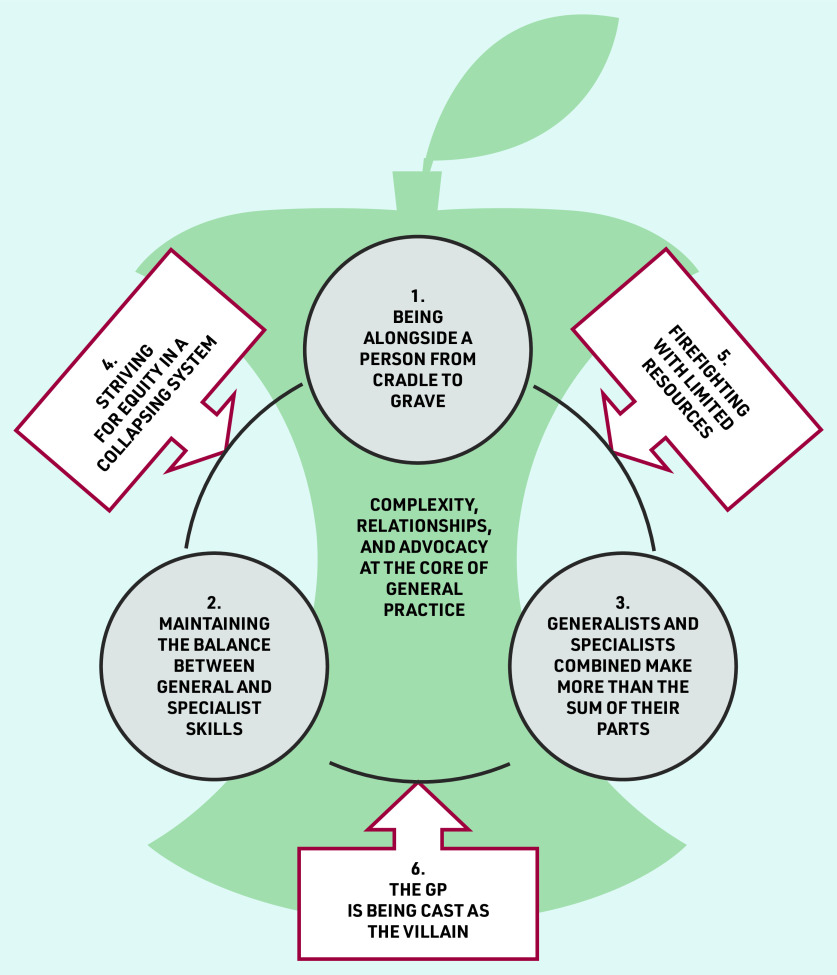
*Storyline of final themes.*

Six themes are presented, incorporated into a storyline(Figure 1):

- being alongside a person from cradle to grave;
- maintaining the balance between general and specialist skills;
- generalists and specialists combined make more than the sum of their parts;
- striving for equity in a collapsing system;
- firefighting with limited resources; and
- the GP is being cast as the villain.

### Being alongside a person from cradle to grave

PCPs described women’s health as complex and not just about reproductive organs; patients rarely came in with one symptom. The unique skill of the GP was to decipher a complex myriad of symptoms, over several consultations, and often with no definitive test:

*‘Women are not just their reproductive organs and their hormonal cycle, and that is the whole essence of primary care, you’re looking after someone as a whole individual, so to try and compartmentalise and separate their hypertension … from their mental health and their reproductive health … is not what primary care is … There’s so much to unpick.’*

(Participant [P]30, GP)

The general practice contributed unique oversight, and each health episode was regarded as a chapter in a person’s life: providing continuity, understanding, and advocacy from cradle to grave. Treatment should be holistic and embedded in a social context. Practitioners described the value of hearing patients’ stories, and not regarding them as a diagnosis, population, or body part.

High-quality practice was underpinned by skills to build relationships:

*‘This is where a strength of primary care* [is] *… knowing people and them trusting you … I think the kind of continuity and that the feeling of your health-home being your GP surgery can’t really be underestimated.’*

(P41, GP)

*‘*[A GP] *understands the narrative of where they’ve come from, has seen them at very difficult times …* [One of my patients] *is currently having some really significant mental health problems … I’ve seen her at a time when she was very capable, and competent and well. I see her in a very different way I think to somebody who would see her now as she is — quite unwell.’*

(P26, GP)

Practitioners described points of communication where solid relationships unlocked valuable information, and how missing important landmarks could be detrimental to wellbeing. A strong relationship was *‘more fruitful longer term’* (P35, GP) and worth the time investment, particularly at first contact:

*‘I had a lady who’d come in for a smear and … we discovered that she’d fled from a domestic violence relationship, and she was living in a caravan, and she was getting really threatening messages … when you have got the time, you can treat them holistically and look at every aspect as a person rather than just honing in on the clinical.’*

(P38, practice nurse)

### Maintaining the balance between general and specialist skills

Practitioners described the challenge of maintaining a balance between generalist and specialist skills, the benefits of specialist knowledge, and the associated risk of deskilling. Practitioners described a tendency for those with a special interest to become the go-to-person, creating a risk of a) losing skills outside a special interest, and b) losing devolved skills:

*‘When you know that there’s somebody else who can do that job better than you … I can probably divert away from me to her so that the patient gets the best care possible … actually maybe I’m not learning as much as I should, or taking as much ownership of women’s health as I should … Over the years you can just sort of completely deskill.’*

(P22, GP)

Expertise within primary care gave the benefit of reducing referrals. Specialist skillsets could be held within different parts of primary care (such as local practices, primary care networks, or community clinics), with different advantages and risks. For example, specialist skills retained in-house brought the opportunity to upskill others, but this training role increased workload:

*‘You’re never going to know everything … When you look at an experienced GP … that takes a lot of years to become like that and everything changes in medicine very quickly, so I think the special interest thing is very good ... I then become a point of contact …* [which] *saves them doing a referral.’*

(P35, GP)

Some felt that the expectation that women prefer to be examined by women fed into the cycle of deskilling male practitioners, significantly increasing the workload of female colleagues:

*‘*[My] *clinical list is very different to my 45-year-old-plus male colleagues … I don’t like the fact that what has shoehorned me into women’s health is being a woman rather than an active choice … Do I find it rewarding? Yes, but I am absolutely aware that it did happen through default by my demographic … women’s health should be everybody’s issue.’*

(P30, GP)

While recognising that specialism could bring advantages, GPs described a hidden workload from devolving care while retaining overall accountability, as supervising others and working within an extended team added complexity:

*‘*[Practitioners with specific skills] *might be more likely to diagnose a chest infection than a deterioration of heart failure because they don’t see as much* [heart failure] *… So I end up carrying that level of risk and uncertainty … There is a hidden complexity and a hidden workload to having extra people doing aspects of your job, and when the buck ultimately stops with you, there is a level of stress that comes along with that.’*

(P26, GP)

### Generalists and specialists combined make more than the sum of their parts

PCPs supported a system where colleagues recognised, valued, and drew on each other’s knowledge and skills, rather than create a division between generalists and specialists. This could be nurtured within specialist women’s centres in the community:

*‘*[Practitioners in secondary care] *perhaps don’t know about managing comorbidities or thinking about the social aspects or challenges that patients may be facing … GPs have a lot more training for consultation skills; how to sort of manage difficult situations, whereas they don’t so much get that kind of training in specialty medicine or gynaecology; so* [learning] *would go both ways.’*

(P44, GP)

Practitioners recognised the value of building links and learning from each other, sometimes in settings where GPs and gynaecologists were co-located:

*‘*[The gynaecologists] *were learning about what we do as a job, and equally we were learning about what they do in their day- to-day job ... With* [domestic] *abuse cases, that’s definitely an example of how there can be an advantage of having GPs involved … knowing what resources were available in the community, and to say, “Have you actually thought about going to the so-and-so centre?”’*

(P44, GP)

Professional networks were highly valued and could help to delay, or even prevent, the need for patient referral. Practitioners drew on formal resources such as NHS Advice and Guidance, and on informal professional networks. Upskilled practitioners in the community could forge links to secondary care and become a training resource. *‘Half- way houses’* (P44, GP), such as community gynaecology were framed as an extension of primary care rather than secondary care in the community:

*‘It’s not taking away from the importance of consultant gynaecology work ...* [but about] *having it sort of embedded in the community, so women don’t have to travel miles and wait to see someone with a bit more expertise … GP specialists managing the core of gynaecology work that was very straightforward and then leaving the consultants to get on with what they really do best.’*

(P20, GP)

Some had noticed an erosion of established links with secondary care, exacerbated by high demand and resource constraints. Service bottlenecks left patients in limbo and contributed to additional workload in general practice. Practitioners faced the challenge of deciding whether, and when, to expedite a referral, and of managing undiagnosed conditions while sustaining relationships:

*‘The patient is waiting 3, 4, 5 months to be seen sometimes, but the patient has still got those symptoms, so what do they do? They don’t pester the hospital, they pester the GP, and you’ve run out of ideas.’*

(P18, GP)

### Striving for equity in a collapsing system

Practitioners were aware of variable health needs and deeply concerned about patients who might *‘fall under the radar’* (P34, GP).

Some of the potential access barriers are illustrated in Box 1. As such, advocacy was at the core of general practice. Some described impractical distances, or unaffordable long waits for patients. Practitioners worried about people delaying care because they did not want to bother the GP, might not define themselves as ill, or because of more pressing priorities:

*‘Ovarian cancer is the one that people worry about, and unfortunately, we found in lockdown that we’ve had a few quite late diagnoses because women didn’t come in and sort of felt that they shouldn’t be bothering us, which is really sad, because we were open.*

**Box 1.** Illustrations of potential access barriers for patient groups

**Patient group**	**Example quote**
**Homelessness and unstable housing**	*‘I think there are always challenges related to people who don’t have stable housing because they’re there one minute and not there the next, so following things through can be tricky.’* (P42, GP)
**Sex workers**	*‘I did work in a sexual health clinic ...* [Sex workers’] *needs are extremely high … they’re vulnerable ... I think a lot of those women probably have just fallen under the radar and just kind of get on with it … I honestly think often you don’t even see those women to be honest ... they’re not even getting to us as GPs.’* (P34, GP)
**Travelling community**	*‘Those more difficult-to-reach patients are from the travelling community and that’s partly to do with their transient nature, if they’re moving around, and ... just trying to communicate with that community can be a bit more difficult: sometimes the levels of education and literacy are lower, and letters that we send out, people might struggle to manage and it’s trying to find different ways to reach out.’* (P10, GP)
**Asylum seekers and migrants**	*‘Vulnerable migrants ... they’ve got possible trauma, there’s the cultural belief systems about how their health should be sort of addressed and then there’s just the other like social-economic things of being able to get to your GP and advocate for yourself.’* (P34, GP)
**Translation needs**	*‘When you’re having to use an interpreter ... It’s, “yes”, “no”, so it’s more closed-ended questions so that is a barrier within itself ... Whereas if you have somebody that speaks English or you have somebody that you can speak with them fluently in another language, it flows more like a story, the history does, because you’re getting to know more information from them and you’re reading in between the lines.’* (P36, clinical pharmacist)
**Health literacy or learning support needs**	*‘Some people may not have a great education in understanding how the body systems work ... People aren’t even aware sometimes how many orifices they have below, and you have to explain, and you’ll say ... the term, “bleeding”, “Where from exactly?” “I don’t know”.’* (P16, advanced nurse practitioner)
**Children and adolescents**	*‘I think teenagers, often they’re just grinning and bearing it too to be honest with you, particularly, whether it be contraception or really heavy periods. It always breaks my heart when they’re coming in and they’re like, “I’m flooding at school” … or, “my periods are so painful”.’* (P34, GP)
**Older patients**	*‘I suspect that* [older people] *struggle more to get appointments than the younger people, because the younger people tend to be a little bit more pushy, they can access things online and so on; whereas the older population that have probably got much more needs, are going to struggle a little bit more with that, and then when they do come in, it often becomes very complicated, a long consultation with lots of different issues and things will then get pushed to the next consultation.’* (P27, GP)
**Trans people**	*‘The NHS registration forms that patients complete when they join a practice, the titles are Ms, Mr, Misses,* [but] *if they’re Mx, we need to know those things so that we can empower and educate here … The receptionist may need to know: like somebody presents as male asking for a smear and ... they need to know not to question* [and therefore make] *the patient feels embarrassed.’* (P28, GP)
**Served a prison sentence**	*‘We’ve got people that have just come out of prison and things like that; they haven’t got access to the internet; they don’t always have mobile phones ... There’s only so much you can do without going and actually knocking on their door and dragging them ... There must be a huge group of people that aren’t even registered with GPs.’* (P32, advanced nurse practitioner)
**Domestic violence**	*‘Patients who are experiencing domestic abuse ... Trying to be able to actually get to a clinician and see them and speak to them and not have your partner breathing down your neck is actually quite hard to do at times. But also, sometimes we find that patients who are in an abusive situation have other factors going on.’* (P44, GP)

*P = participant.*

Practitioners described a deeply entrenched devaluing of women’s health that could be a barrier, recognising that cultural normalising of women’s health concerns meant that women did not always readily come forward for support:

*‘There’s so much historical sort of cultural thing of women should just put up with this, it’s just natural, it’s just something that they have to endure, and a lot of them come late, and they’ve tried to battle this, and they’ve come when they’re really in a pickle, and their relationship’s on the rocks, and their job’s suffering, and you think, “how many other conditions do people wait this long before they come to the doctors?” Not many.’*

(P8, GP)

Practitioners felt that women might be wary of attending because of previous experiences. Some described the value of the media in breaking down barriers and tackling stigma:

*‘30- or 40-year-old ladies will say, “I always, always really suffer with my periods, but I was always just told it was normal, it’s just part of you know what women go through” … I’m not sure if it’s because of* [the] *kind of things on TV or ... in the media promoting women’s health … trying to break this idea that it’s normal for periods to be so painful or so heavy that it’s debilitating.’*

(P21, GP)

### Firefighting with limited resources

Although practitioners recognised that relationship-based care was integral to general practice, resource constraints challenged this core value, and created the risk of transactional consultations. Strategies for retaining the relational aspects were harder with squeezed resources:

*‘The patient experience of the doctor not caring,* [I] *don’t think directly reflects whether or not the doctor actually cares, it reflects the skillset they have, the time that they have, the resources that they have, and the fearfulness of just opening the can of worms and simply not having the capacity to actually deal with it, so inadvertently they shut it down, and it’s not because they don’t value it.’*

(P30, GP)

Practitioners did their utmost to work with available resources, but had to balance their ideals against what resources would allow. This could feel uncomfortable and presented additional professional and personal distress:

*‘Personally, I think it’s really horrible having to like rush through something … I already know that I can’t do everything for you in 10 minutes, which isn’t always like a nice feeling for me, because we want to be able to help … Who knows when they’ll be able to get an appointment again.’*

(P35, GP)

Some described the pressure of working under unrealistic expectations of dealing with women’s health in a 10-min appointment, and the risk of missing something critical. This could threaten professional identity:

*‘I fit coils and, as ridiculous as it sounds and I’m slightly embarrassed to say it, that even sometimes when I see that somebody needs a smear, I don’t do it because I’m already doing a consultation in half an hour that’s going to take me 45 minutes … So I will book them in with somebody else for another date; and that just feels massively inefficient … actually wrong.’*

(P14, GP)

Some felt that resource constraints had an impact on job satisfaction, recruitment, and retention, particularly in areas of socioeconomic deprivation where health and social care needs were additionally complex. Practitioners recognised that lengthier appointments would reduce access for others or put off those who could not wait if clinics overran. Some practitioners avoided dealing with more than one health issue in an appointment or referred sooner.

### The GP is being cast as the villain

The challenge of limited resources had become harder in the context of *‘media bashing’* (P13, GP).

For practitioners holding patient advocacy as integral to the ethos of primary care, this media assault struck at their very core, with an impact on professional pride and vocation, and put up barriers:

*‘It puts barriers up between us and the patients … The papers are all running with it … “You’re earning this amount of money … ” You just think, why do you work against us? ... The job’s hard enough … It just feels so unfair …* [People previously] *respected us, respected our job, our knowledge, that has gone by-the-by now.’*

(P24, GP)

Participants asked for a more balanced conversation in the media, and society, about health complexity and treatment risks. Some felt that media representation could undermine opportunities for shared decision making, and that patients were not always fully apprised of safety issues:

*‘Menopausal symptoms can be quite vague … I would automatically want to look into thinking about other more sinister causes … As GPs we’re always their first point of contact from anything that happens in the news …* [patients come in saying] *… “I do have aches and pains and my bones must be bad, please can you start me on* [hormone replacement therapy {HRT}]*?” and then you kind of have to backtrack that expectation a little bit and say, “actually well … it’s a balance of risk and benefits”.’*

(P35, GP)

Practitioners valued the positive aspects of patients becoming self-advocates; however, this could sometimes lead to adversarial consultations:

*‘There’s a massive challenge … we’re seeing huge numbers of women who are coming, often angry … believing that what the media’s telling them, that doctors are refusing HRT … they’re coming kind of wound up to have a fight … convinced by the current media story that everything is about menopause and that doctors are denying people HRT … Many of us feel we’re being kind of forced to* [prescribe] *by the sort of media zeitgeist … There needs to be a more balanced conversation.’*

(P42, GP)

Some found it uncomfortable if asked to provide treatment against guidelines or professional judgement:

*‘I’m listening to you, but I can’t give you what you want because it is not safe.’*

(P22, GP).

Practitioners wanted patients to understand that GPs have expert knowledge and are advocates for patients. Practitioners felt that the media perception about GPs *‘on the golf course.’* (P41, GP) should be addressed. Some felt that working in general practice had become unattractive and practitioners were exiting the profession:

*‘People need to fall in love with being a GP again … I don’t think it looks attractive to people, so the whole thing about retention and more recruitment even if we were allowed to have more, I don’t think it looks like something people want to do at the moment, and I think we need a wholescale PR* [public relations] *campaign.’*

(P41, GP)

### Storyline of final themes

Figure 1 depicts the core value of general practice as being alongside a person from cradle to grave (theme 1); it is encompassing practice that recognises and responds to complex needs, and advocates for patients.

Quality health care is built on human connection, and building and holding longitudinal relationships. PCPs face the challenge of maintaining a balance between general and specialist skills in the context of a rapidly evolving field of knowledge (theme 2). The key to maintaining this balance is acknowledging differential skills, learning from each other, and knowing that working together (rather than against each other) provides more than the sum of its parts (theme 3). PCPs face external challenges to their advocacy role and are acutely aware of barriers to access (theme 4). Resource constraints place additional strain and create the risk that patient–practitioner relationships become transactional rather than relational (theme 5). This can lead to personal and professional consequences that have an impact on care. PCPs feel cast as the villain (theme 6), despite the heavy value they place on advocacy as integral to their role. This external pressure can have far-reaching repercussions if PCPs feel pressurised to provide treatment that they do not feel is in their patients’ best interests.

## DISCUSSION

### Summary

The current findings show that relationships and advocacy are at the core of women’s health, and highlight threats to these values and skills. The authors heard reflections on the re-configuration of services, evolving models of care, enabling special interests without deskilling others, and optimising education and shared benefits.

GPs told us how they strive to deliver equity for those facing difficulties in accessing care. Resource constraints create the risk that relationships became transactional rather than relational. This work occurs in the context of service challenges, resource constraints, and negative media coverage that affects PCPs wellbeing and work.

### Strengths and limitations

The current study uniquely explores PCPs perspectives on how to meet the aspirations of the Women’s Health Strategy.^9^ The remit of this study was broad, enabling the introduction of unanticipated ideas that may be transferable beyond women’s health care. The authors spoke to 46 PCPs from a range of socioeconomic settings. Most participants were female, and additional male PCPs could offer a valuable sample extension.

The strength of qualitative research is its interpretation. Braun and Clarke suggest that qualitative researchers make a *‘subjective judgment about when to stop’* collecting data when themes are coherent (yet nuanced), and *‘convey something important’*.^11^ They argue that data saturation is aligned with positivism and *‘deeply problematic’* for reflexive thematic analysis. Information power, proposing that the more information a sample holds the fewer participants are needed, may be a more useful strategy.^12^ As here the intention was to explore a broad topic using a mixed sample, it was anticipated that a large sample was needed.

Statistical generalisability is not a relevant standard to judge qualitative research.^11^^,^^13^^,^^14^ The value of qualitative research is to generate ideas that have the potential to transform how the audience sees or interacts with the world outside the research context.^14^ This alternative form of generalisability is described as ‘theoretical’, ‘analytical’, ‘conceptual’, or ‘idiographic’.^11^^,^^13^^,^^14^

### Comparison with existing literature

The Women’s Health Strategy for England^9^ indicates that women’s healthcare delivery needs to improve. Qualitative research highlights the distress inherent in navigating complex healthcare systems for stigmatising symptoms. For example, intimate partner violence,^15^ endometriosis,^16^ polycystic ovary syndrome,^17^ lupus,^18^ and maternity care,^19^ among others. The Ockenden review^20^ and Cumberlege^21^ report highlight the potential harm from not hearing patients’ voices, and PCPs can offer valuable insight for improved care.

To improve women’s health care, more needs to be understood about the wider structural frameworks of general practice. General practice in England is increasingly delivered by multidisciplinary teams and in collaborative local groups of GP practices under the primary care network contract. Although potentially offering resilience and service diversity, the authors’ findings in the current study suggest that these changes bring new challenges, including in managing devolution of care and supervision of other team members, and the potential impact on continuity.

The Women’s Health Strategy^9^ goes beyond identifying challenges and recommends the development of women’s health hubs to bring together services and expertise for women. Hubs offer opportunities to make changes and improve how women are supported. However, how these are implemented, where they are located, and by whom they are staffed is less clear.^22^ General practice is ideally situated to support women throughout the life course.^3^ Any changes will need careful evaluation to ensure that they do not contribute to already significant health inequalities.^4^^,^^23^

Women’s health hubs are being implemented, with an interim evaluation that focuses on perspectives from within the hub structure, including opportunities for reciprocal learning, and the balance between opportunities for upskilling and risks of deskilling.^10^ The current study adds reflections on the risks of taking women’s health out of general practice, which warrants further careful attention.

Finally, the current study’s findings resonate with reports about the impact of negative media representation on GP recruitment^24^ and retention,^25^ and highlight the impact on staff morale and repercussions for care. The authors heard about moral tensions and distress as PCPs strived to work and deliver care within a constrained system, while being vilified in the press.

### Implications for research and practice

There is commitment to innovate and to deliver excellence in women’s health care within primary care. There is recognition of the need for, and value of, adapting ways of working, and supporting new ways of collaborating with community and specialist services. Developing specialist roles, expertise, and bespoke services can foster wellbeing for staff and could support retention. The benefits for knowledge development, service delivery, and patients that accrue from closer collaborations are likely to be valued and reciprocal. However, this warrants close evaluation, in terms of the impact, both anticipated and unintended. Care is needed to ensure that service configuration changes do not result in clinician deskilling or making services inaccessible for some patients. Care is also needed when evolving services so that the core aspects of general practice are not devalued, including relationship-based longitudinal knowledge of individuals, families, and communities. GP teams are well placed to advocate for their patients, including commitment to seeking equitable care, and these skills and specialist knowledge should be actively recognised, valued, and nurtured.

Further research would usefully explore the interface between specialist interests and services, GP retention and sustainability, how and where these services ideally sit, and actively consider and evaluate both the intended and unintended consequences of these.
